# Comprehensive In Silico Analysis of Retrotransposon Insertions within the *Survival Motor Neuron* Genes Involved in Spinal Muscular Atrophy

**DOI:** 10.3390/biology11060824

**Published:** 2022-05-27

**Authors:** Albano Pinto, Catarina Cunha, Raquel Chaves, Matthew E. R. Butchbach, Filomena Adega

**Affiliations:** 1 Laboratory of Cytogenomics and Animal Genomics (CAG), Department of Genetics and Biotechnology (DGB), University of Trás-os-Montes and Alto Douro (UTAD), 5000-801 Vila Real, Portugal; albanopinto96@gmail.com (A.P.); catarinaecunha@hotmail.com (C.C.); rchaves@utad.pt (R.C.); 2 BioISI-Biosystems & Integrative Sciences Institute, Faculty of Sciences, University of Lisboa, 1749-016 Lisbon, Portugal; 3 Division of Neurology, Nemours Children’s Hospital Delaware, Wilmington, DE 19803, USA; matthew.butchbach@nemours.org; 4 Department of Biological Sciences, University of Delaware, Newark, DE 19716, USA; 5 Department of Pediatrics, Sidney Kimmel College of Medicine, Thomas Jefferson University, Philadelphia, PA 19107, USA

**Keywords:** spinal muscular atrophy, transposable elements, retrotransposons, genome dynamics, *SMN1*, *SMN2*

## Abstract

**Simple Summary:**

Transposable elements are DNA sequences that can move throughout the genome. They play essential roles in gene regulation and function. Spinal muscular atrophy (SMA) is a leading genetic cause of infant mortality worldwide. Since transposable elements have been linked to other genetic diseases, we examined the genomes from SMA patients as well as healthy genomes for the presence of transposable elements. We identified distinct transposable elements that may impact gene expression by affecting promoter activity or transcriptional termination of the *SMN* genes. These elements within the SMA genes may play key roles in understanding this early-onset neurodegenerative disease as well as how transposable elements can impact gene expression. Understanding the roles of transposable elements in SMA may provide key insights into other neurodegenerative diseases.

**Abstract:**

Transposable elements (TEs) are interspersed repetitive and mobile DNA sequences within the genome. Better tools for evaluating TE-derived sequences have provided insights into the contribution of TEs to human development and disease. Spinal muscular atrophy (SMA) is an autosomal recessive motor neuron disease that is caused by deletions or mutations in the *Survival Motor Neuron 1* (*SMN1*) gene but retention of its nearly perfect orthologue *SMN2*. Both genes are highly enriched in TEs. To establish a link between TEs and SMA, we conducted a comprehensive, in silico analysis of TE insertions within the *SMN1*/*2* loci of SMA, carrier and healthy genomes. We found an Alu insertion in the promoter region and one L1 element in the 3′UTR that may play an important role in alternative promoter as well as in alternative transcriptional termination. Additionally, several intronic Alu repeats may influence alternative splicing via RNA circularization and causes the presence of new alternative exons. These Alu repeats present throughout the genes are also prone to recombination events that could lead to *SMN1* exons deletions and, ultimately, SMA. TE characterization of the SMA genomic region could provide for a better understanding of the implications of TEs on human disease and genomic evolution.

## 1. Introduction

Transposable elements (TEs) are interspersed repetitive DNA sequences with the ability to mobilize in the genome. This mobility is mediated by element-encoded proteins such as DNA transposase or reverse transcriptase and occurs within the genome of virtually all walks of life, including prokaryotes, unicellular and multicellular eukaryotes and even large DNA viruses [1,2]. TEs can be divided in two major classes, retrotransposons (class 1) and DNA transposons (class 2), based on their mechanism of transposition [3,4,5]. Retrotransposons have a “copy-and-paste” mobilization mechanism where an RNA intermediate is reverse-transcribed into a cDNA copy that is integrated elsewhere in the genome [3,6,7]. On the other hand, class 2 transposons mobilize via a DNA intermediate associated with a transposase [3,4].

Retrotransposons are divided in two major subclasses based on the presence or absence of a long terminal repeat (LTR) sequence. Long and short interspersed nuclear elements (LINEs and SINEs, respectively) comprise the two types of non-LTR retrotransposons. The only active, autonomous TE family in humans is the LINE-1 family, although most of these sequences are no longer mobile due to various forms of rearrangements, mutations and 5′-truncation [3,8,9]. SINEs, on the other hand, are not autonomous but contain a 3′ tail homologous to a LINE [4,5] used to “parasitize” the enzymatic machinery of LINEs and thus, to mobilize [4]. In this way, active SINEs, such as the primate-specific Alu elements, are completely dependent on the endonuclease and reverse transcriptase of L1 elements for genome propagation [3,6,9]. Within the human genome, Alu sequences are divided into subfamilies based upon sequence variations and accordingly to their evolutionary age, with (J) standing for the oldest Alu subfamily, (S) for the intermediate and (Y) for the youngest subfamily [10]. Thus far, only some AluY subfamilies are retrotransposition-competent in the modern human genome [9].

Initially considered inert remnants of evolution and so called genomic parasites, TEs are now recognized as important players in genomic evolution, genome organization and gene regulation—due primarily to the advances in genome sequencing and better analysis tools Once co-opted by the host genomes, TEs provide important sources of new regulatory sequences that can act as alternative promoters, tissue specific enhancers, splice sites, polyadenylation signals, insulators, termination sites and transcriptional factor binding sites, thereby altering nearby gene expression in cis [6,11,12,13,14,15]. TEs can also impact mammalian development and evolution through their domestication or the domestication of their proteins. The human genome contains around 50 to 150 genes that are probably domesticated or derived from TEs [6,16,17]. Exonization of intronic TEs—i.e., intronic TEs that are incorporated into exons of coding or noncoding transcripts—also gives these elements the ability to expand the mammalian transcriptome and proteome. Intronic Alu elements are particularly prone to be captured as alternative exons through cryptic splice sites residing within key positions of their sequences [3,6,13,14,18].

While TEs play a beneficial role in genome evolution, their presence can also be detrimental to the host and cause several problems to normal gene expression and to genome organization, stability and integrity [6,8,11,19]. The ability of TEs to transpose is the main mechanism associated with TE-induced diseases, with TE insertions into genes acting as insertional mutagens and interfering with gene function [6,17,19]. Not surprisingly, the more actively propagating TE families in the human genome are the ones responsible for the onset of some of these diseases, namely (and in order of prevalence) Alu, L1 and SVA families [17]. De novo germline and somatic TE insertions disrupting normal gene function have been implicated in several human diseases, among them neurologic disorders and cancer [8,9,11,19]. Alu elements and other TE families are also capable of promoting chromosomal rearrangements between the highly homologous regions dispersed by related TEs at distant genomic positions resulting in small and large-scale deletions, duplications and inversions [3,20,21]. Another way TEs can pose a problem to the host genome stability and coding potential is through their ability to influence gene splicing. Alu elements—in particular inverted Alu repeats located within introns—are capable of influencing mRNA splicing, resulting in the formation of circular RNAs and altered splicing patterns [8]. This may result in nuclear retention of RNA and loss of protein-coding potential, making TEs potential agents of disease-causing events [8,22].

Attention to the contribution of TEs to neurodegenerative diseases has been rising in the last few years [23]. Spinal muscular atrophy (SMA) is an early-onset, autosomal recessive neurological disease characterized by degeneration of motor neurons in the anterior horn of the spinal cord and brainstem nuclei [24,25]. This motor neuron degeneration leads to progressive muscle weakness and atrophy. This neurodegenerative disease affects approximately 1/6000 to 1/10,000 individuals and is the most common inherited cause of childhood mortality [26]. The carrier frequency for SMA is highly variable between populations, ranging between 1/25 to 1/50 [26,27]. Most cases of SMA result from a complete loss of *Survival Motor Neuron 1* (*SMN1*) but retention of the paralogous *Survival Motor Neuron 2* (*SMN2*) gene [26,27,28]. *SMN1* and *SMN2* are nearly identical except for 20 single nucleotide differences, with the C to T transition in exon 7 (c.840C > T) being the most functionally relevant difference [29]. *SMN1* is located in a highly unstable region of the large arm of chromosome 5 (5q13.2), a region of the genome that is enriched with repeated sequences, pseudogenes and transposable elements [30,31,32]. It has been hypothesized that TEs may be the cause behind the instability of this region, given the ability of this mobile genetic elements to promote genetic instability and large chromosomal rearrangements observed in 5q13.2 [30,31]. *SMN1* and *SMN2* are both highly enriched in TEs—especially Alu and L1 repeats—spanning both genes [33]. The high abundance of Alu elements and other transposable elements in *SMN1* introns have an impact on the regulation of the splicing patterns as two Alu elements can give rise to new alternative SMN exons as well as on circularization events of SMN RNA that result from inverted Alu repeats [33,34,35]. Such accumulation of Alu repeats in *SMN1* introns also makes this gene prone to deletion events caused by Alu/Alu recombination events [36,37,38].

Prior studies on the organization of TEs within the *SMN1* and *SMN2* (*SMN1*/*2*) genes, have only used the reference gene sequence in their analysis [34]. As some TE families are actively transposing and increasing in copy number within the human genome, TE insertions may not be present in the reference genome assembly. Analysis of individual genomes using next generation sequencing (NGS) technologies allows the detection of segregating structural variants within human populations and genotyping of transposable elements in healthy and diseased individuals. In this study, we will compare the location and orientation of TE insertions at the *SMN1*/*2 loci* of whole genome sequences from SMA patients, SMA carriers and healthy individuals. The results of this comprehensive bioinformatic analysis could provide important insights into the potential involvement of TEs in SMA onset as well as help understand the roles of TE dynamics in genome evolution, gene regulation and human disease.

## 2. Materials and Methods

### 2.1. SMA Genomic Sequence Cohort

Our sample database consisted of *SMN1*/*2* gene sequences from 20 SMA carriers, 22 non-carriers and 37 SMA patients obtained from different sources. Genome sequencing data from the “1000 Genomes Project” cohort [39] were selected by their greater than 70% probability of being carriers for SMA based on a Bayesian model described [40] previously (Table 1). Genome sequencing data from the 37 SMA patients and the two healthy individuals have been previously published by the Motor Neuron Diseases Research Laboratory (MNDRL, Wilmington, DE, USA) in collaboration with Illumina, Inc. (San Diego, CA, USA) [41]. The SMA status as well as *SMN1* and *SMN2* copy numbers for the MNDRL cohort were confirmed by digital droplet PCR (Table 2) [42,43,44]. We also analyzed the reference *SMN1* sequence (gene ID: ENSG00000172062, human genome assembly GRCh38, p13) that is available in Ensemble.

### 2.2. In Silico Analysis of Sequencing Data

The raw next generation sequencing (NGS) data obtained were processed and then mapped against *SMN1* reference sequence by Geneious Mapper tool [45] (Geneious Prime version 2020.0.5 software, Biomatters, Ltd., Auckland, New Zealand). The mapping sensitivity was set to medium and the number of iterations was set to 10 times. Geneious Mapper generated a contig of the multiple reads mapped to *SMN1* and a consensus sequence of the mapped reads which was used for predicting TE insertions.

All *SMN1*/*2* sequences obtained and the *SMN1* and *SMN2* Consensus Coding Sequences (CCDS) [46] isoforms in study were screened for TEs insertions using Dfam version 3.1 [47,48]. This search comprised two parts: (1) a search of the sequence against all the Dfam models and (2) a search against the tandem repeat finder (TRF) tool, which is part of the Dfam search method. This tool allows for searching TE insertions in up to 50 kb DNA sequences against Dfam database. The source organism was specified as “*Homo sapiens*” to ensure that the best cut-off was applied to each model thereby ensuring more accurate predictions of TE location and orientation. Overlapping Dfam matches (nearly perfect overlaps) are automatically removed by Dfam so as to remove model redundancy.

All the TE annotated *SMN1*/*2* sequences and CCDS were aligned using Clustal Omega version 1.2.2 multiple sequence alignment (MSA) program [49] (Geneious Prime version 2020.0.5) and the predicted TE insertion sites and subfamilies were compared between all samples. *SMN1*/*2* transcriptional elements and motifs, including the promoter elements and other regulatory sequences were either described previously [50,51] or were computationally predicted by EMBOSS Nucleotide Analysis version 1.1.1 [52] (Geneious Prime version 2020.0.5).

## 3. Results

### 3.1. Transposable Elements and SMN1 Transcription

**Figure 1:**
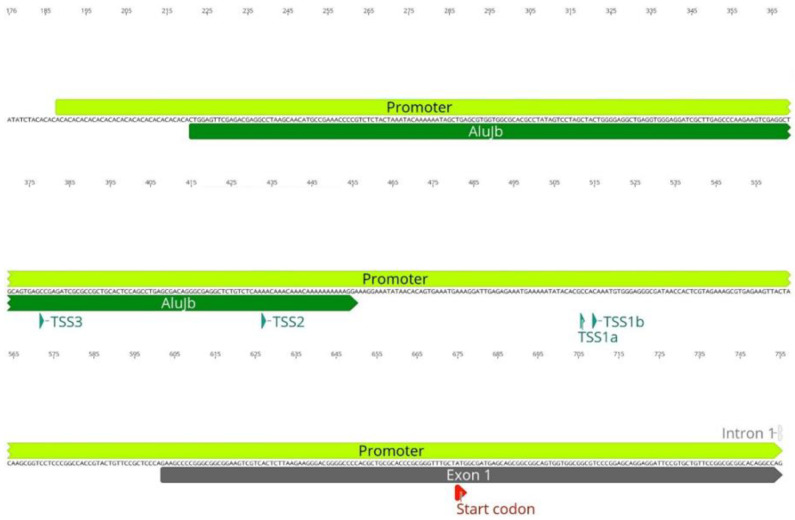
AluJb element within the *SMN1*/*2* promoter region. An AluJb element (represented by a dark green arrow) is inserted inside the promoter region of *SMN1*/*2*, upstream of the canonical transcriptional start site (TSS1a). Transcriptional start sites (TSS), two of them located inside the AluJb sequence, and the start codon are represented by green and red arrowheads, respectively.

**Figure 2:**
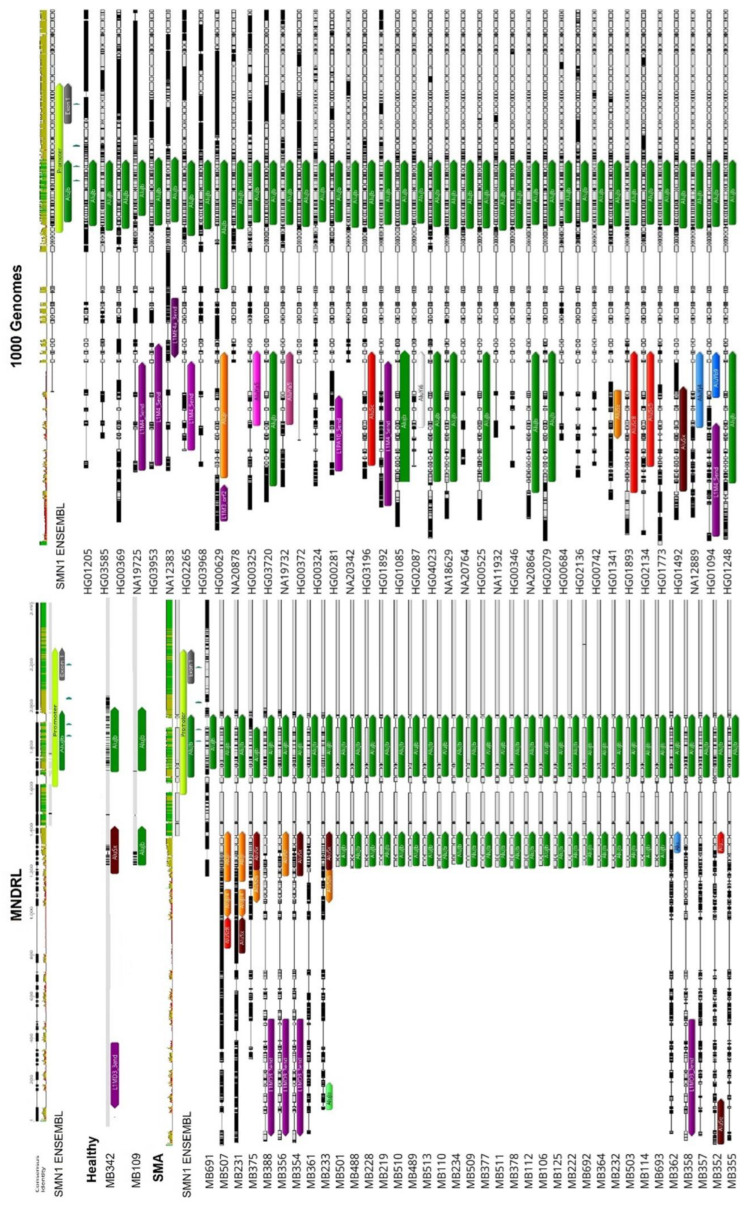
Comparison of the *SMN1*/*2* promoter regions within sample cohort. An AluJb insertion (represented by a dark green arrow) is present within the gene promotor region of all samples. Some polymorphic insertions were detected upstream of the promotor region and the 5′UTR; there were no connections, however, between these polymorphic insertions and SMA phenotype.

As previously stated, *SMN1* and *SMN2* are highly enriched in TEs, including in key regions for gene transcription, such as the promoter and terminator regions. Our analysis of the *SMN1*/*2* promoter identified insertion of an AluJb repeat inside the promoter regions of both genes (Figure 1). This AluJb sequence harbors several transcription regulatory motifs upstream of the most used transcription start site (TSS1) including a fetal transcription start site (TSS2) and another transcription start site (TSS3) (Figure 1). The EMBOSS Nucleotide Analysis tool identified many other regulatory motifs within this Alu element sequence (Figure 1). Our analysis showed that all samples in study, SMA patients, carriers, non-carriers, healthy and the reference *SMN1*/*2* sequence from Ensembl exhibit this Alu insertion in the promoter as well as harbor the two alternative transcriptional start sites and remaining regulatory motifs (TSS2 and TSS3) (Figure 2).

**Figure 3:**
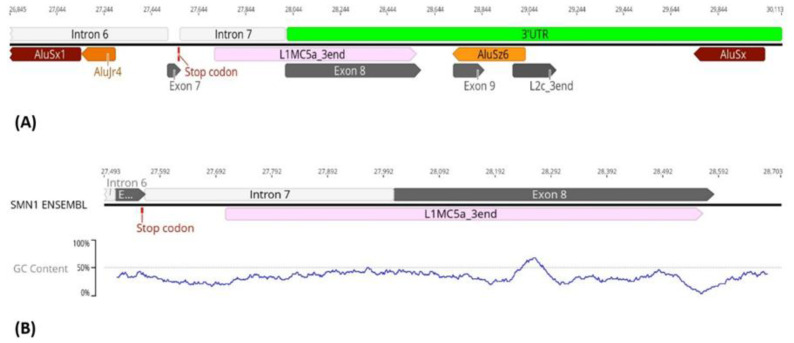
Identification of TEs within the *SMN1*/*2* 3′UTR. (**A**) *SMN1*/*2* 3′UTR region is enriched in transposable elements (represented in colored arrows). Exon 8 is considered part of the 3′UTR region of the gene. An L1MC5a element (pink arrow) is inserted in *SMN1*/*2* exon 8 (grey arrow). (**B**) A large L1 insertion (represented by a colored arrow) was detected in *SMN1*/*2* last canonical exon, exon 8 (represented by a grey arrow). G + C analysis of the region showed a general lower G + C content in this region compared with the adjacent gene regions, partially explaining how a L1 insertion occurred in this region.

**Figure 4:**
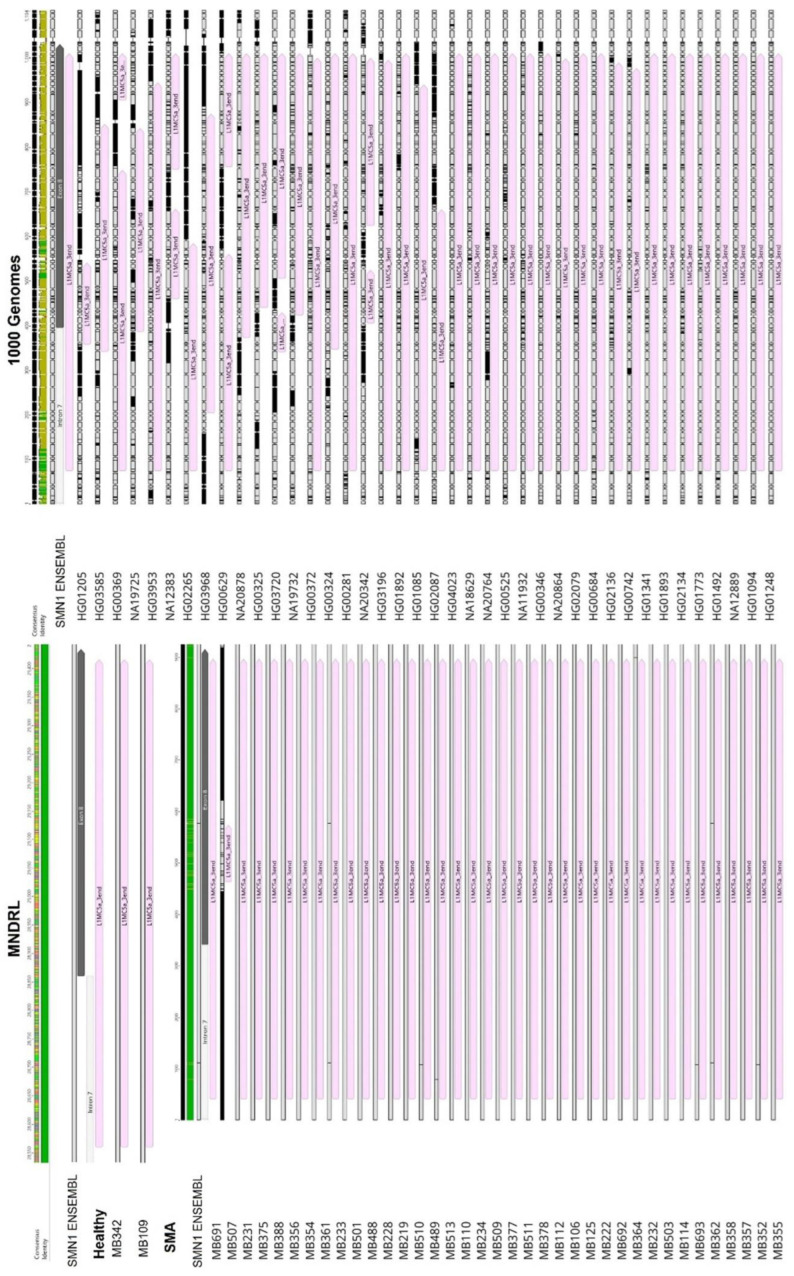
Comparison of *SMN1*/*2* exon 8 within the sample cohort. All samples in study, independently of disease status, exhibit the L1 insertion (represented by a pink arrow) inside exon 8 (represented by a grey arrow) suggesting that the L1 element inserted in exon 8 is indeed fixed in the population and that it has a biological role in *SMN1*/*2* regulation.

Interestingly, the region upstream of *SMN1*/*2* promoter exhibits differences in TE insertion sites and subfamilies present between samples. We hypothesize that the untranslated regions may be subjected to less evolutionary pressure thereby allowing more diversity in TE insertions. With respect to the gene region downstream of exon 7, our first analysis of *SMN1*/*2* gene reference sequence obtained from Ensembl showed several TE insertions belonging to various subfamilies (Figure 3A). The last canonical exon (exon 8) of *SMN1*/*2* is located within the terminator region of the gene and primarily serves as the 3′UTR region of the gene [34,53]. We detected a L1 insertion within this exon. This is a truncated L1 insertion corresponding to the 3′ end of a L1MC5a subfamily retrotransposon (Figure 3B). Our analysis of the remaining samples in study showed that independently of being SMA patients (exhibiting *SMN1* deletion), SMA carriers, non-carriers or healthy genomes, all samples show this L1 insertion inside exon 8 (Figure 4). Given that exon 8 serves as the 3′UTR region of the gene and that the L1 insertion within exon 8 is present in all samples regardless of disease status, we argue that the insertion of this retrotransposon in the 3′UTR region arises from a domestication event which gave this gene a novel, alternative terminator.

**Figure 5:**
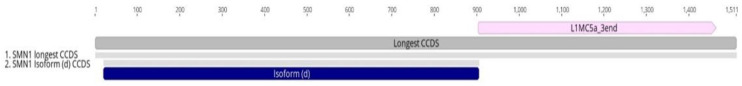
Identification of TEs within the *SMN1* Consensus Coding Sequences (CCDSs). *SMN1* longest isoform CCDS represented in grey on top has a L1MC5a element (represented by a pink arrow) inserted in its sequence responsible for the extension of the CCDS. Contrarily, *SMN1* most common CCDS, isoform (d; represented as a blue bar), is shorter and does not have any TE insertion in its sequence. Both sequences show 100% sequence identity within the overlapped region. We also observed the presence of a L1MC5a element within the longest CCDS for *SMN2*.

To determine whether the L1MC5a element inserted in exon 8 is being expressed in the *SMN1* coding sequence, we conducted a deeper analysis of the *SMN1* CCDS. Analysis of the longest *SMN1* transcript (GenBank: BC062723.1) in Dfam showed a L1 element at the 3′ end of the CCDS, indicating that these complete SMN1 transcripts have an imbedded TE sequence derived from the L1MC5a insertion in exon 8 (Figure 5). Interestingly, the predominant transcript of *SMN1*, isoform d (CCDS34181.1), does not contain this L1 insertion (Figure 5). These results suggest that the alternative terminator function of the L1 element in exon 8 is correct since the L1 insertion is only detected in the longest isoform of *SMN1* transcripts and effectively functions as an alternative transcription terminator to the canonical *SMN1* terminator.

Since *SMN1* and *SMN2* share extensive sequence homology and nearly identical TE insertional patterns, we analyzed *SMN2* transcripts and found that the longest isoform (BC000908.2) contains the same L1MC5a insertion in a similar 3′ location to that seen in the longest *SMN1* transcript (results not shown). Similar to *SMN1*, the remaining and more common *SMN2* transcripts isoforms (d, a, b and c) do not exhibit any L1 insertion in their sequence (results not shown).

### 3.2. Transposable Elements and Alternative Splicing by Exonization

**Figure 6:**
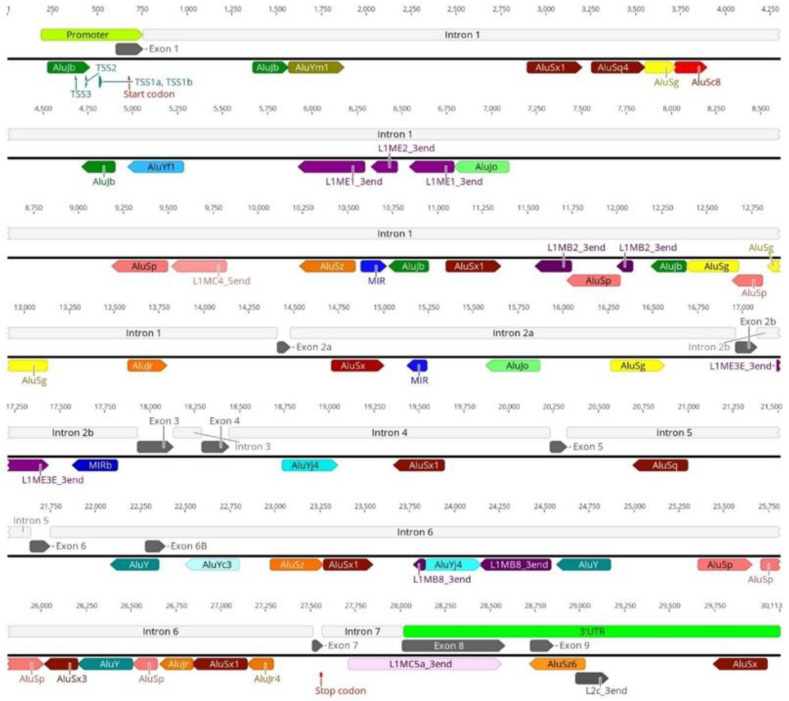
Identification of TEs within the reference *SMN1*/*2* gene sequence. *SMN1*/*2* exons are represented by grey arrows and introns by white boxes. *SMN1*/*2* promoter is represented by a green box and other regulatory motifs by green arrows. Start and stop codons are represented by small red arrows. Transposable elements position and orientation is indicated by colored arrows, with the direction of the arrow indicating the orientation of the repeat element.

**Figure 7:**
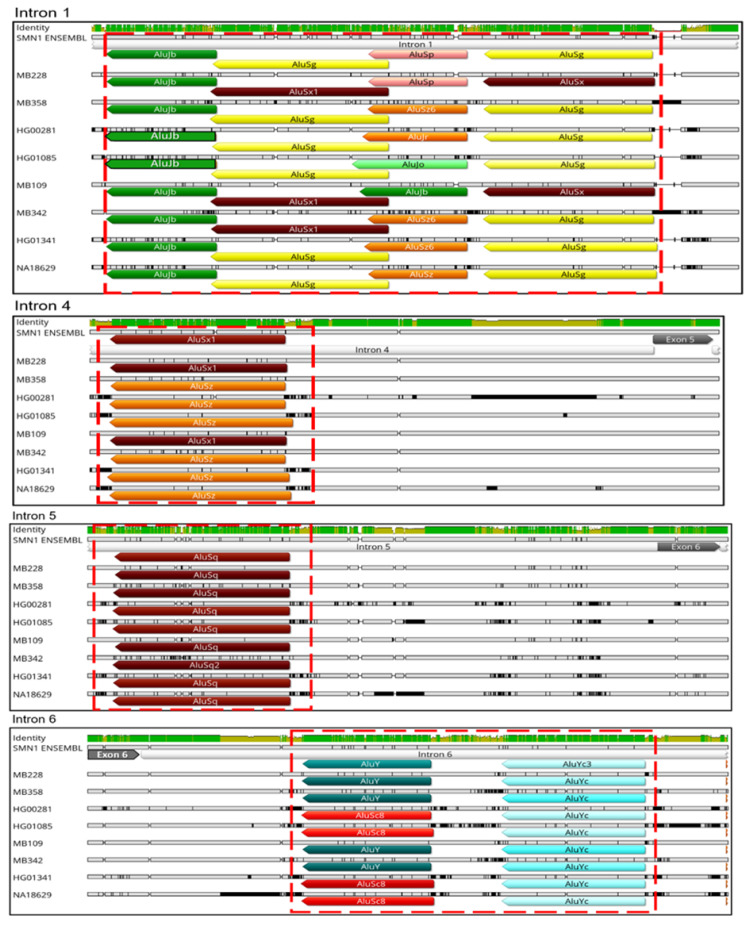
Identification of Key Alu repeats involved in RNA circularization events within *SMN1*/*2*. Comparison between key Alu repeats involved in *SMN1*/*2* circularization events (inside the red boxes) located in introns 1, 4, 5 and 6. Independently of disease status, a conservation of position and orientation of the Alu insertions is visible. SMA patient samples (MB228 and MB358); SMA carriers (HG00281 and HG01085); healthy samples (MB109 and MB342) and Non-carrier samples (HG01341 and NA18629). Color codes for the arrows: green, AluJb; pink, AluSp; yellow, AluSg; orange, AluSz6; light green, AluJo; dark red, AluSx1; teal, AluY; light blue, AluYc and red, AluSc8.

*SMN1*/*2* introns are highly enriched in Alu-derived repeats with many of them in an inverted orientation (Figure 6). These inverted Alu repeats—mainly AluY, AluJr and AluSx1—span the whole locus but accumulate particularly in intron 1, 2a, 4 and 6. Our analysis of the remaining samples showed that all samples have the same pattern of Alu insertions in *SMN1*/*2* introns with the key Alu repeats involved in the genes’ transcripts circularization being located in the same position and orientation as in the *SMN1* reference sequence obtained from Ensembl (Figure 7).

**Figure 8:**
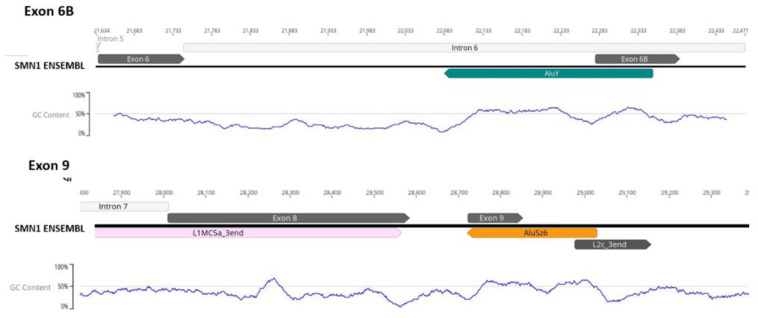
Identification of *SMN1*/*2* alternative exonization events. On top, exonization event of an intronic antisense Alu repeat (represented by a green arrow) that gave birth to alternative exon 6B. Below, another exonization event of an antisense Alu element (represented by an orange arrow) that resulted in the formation of *SMN1*/*2* alternative exon 9. G + C content analysis of both regions shows a higher G + C content in the exonization regions when compared with the surrounding areas, which might have favored Alu insertions and the posterior exonization events.

Another important role of these inverted Alu repeats located in *SMN1*/*2* introns is a complex TE domestication event that gives the genes new coding sequences, in a process termed exonization. Among the several TE families capable of forming new exons, Alu elements are particularly prone to be domesticated as alternative exons [3,18]. It has been estimated that 5% of all alternatively spliced human exons derive from the exonization of Alu elements [54,55]. In *SMN1* and *SMN2*, two alternative exons resulting from exonization have been reported thus far, exon 6B [56,57] and exon 9 [58]. These two exonization events differ in the Alu subfamily involved, as well in the Alu arm involved in the exonization. Exon 6B results from exonization of the left arm of an inverted AluY element and exon 9 originates from the right arm of an antisense AluSz element (Figure 8).

**Figure 9:**
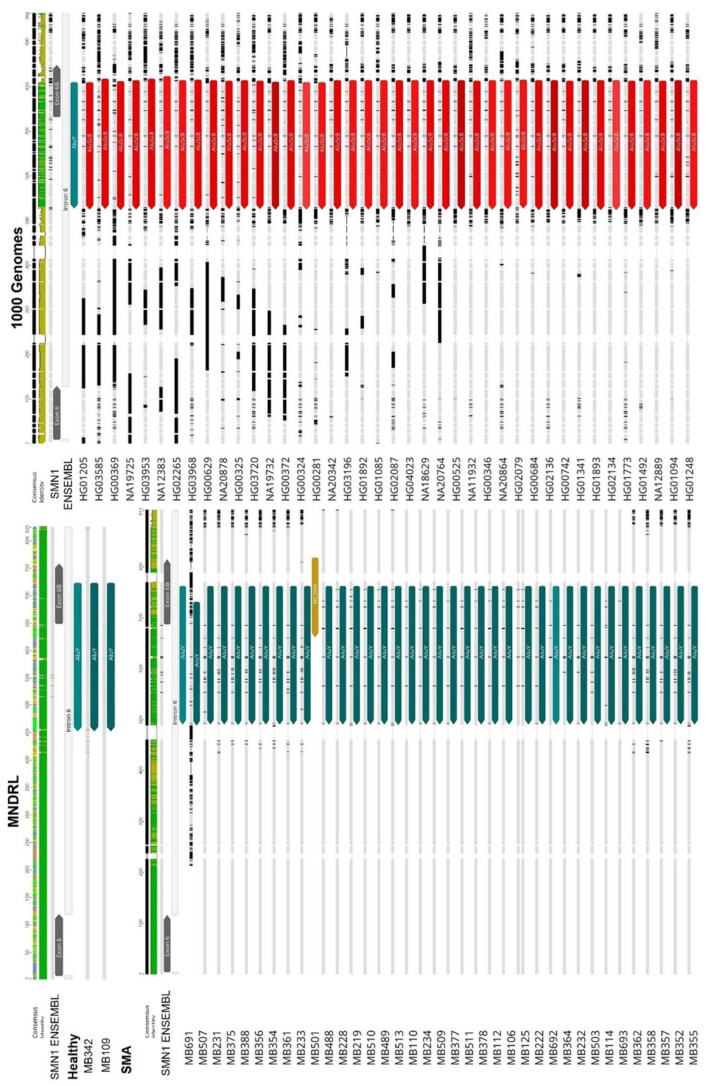
Comparison of the exon 6B region within the sample cohort. An *AluY* insertion (represented by a teal arrow) gives rise to alternative exon 6B in all healthy and SMA samples, including the reference *SMN1* ENSEMBL sequence. Contrarily, an *AluSc8* (represented by a red arrow) insertion is present instead of the *AluY* element in the remaining samples. This Alu insertion difference is most likely the result of low sequencing read depth of the 1000 Genomes Project samples.

We identified some insertional polymorphisms regarding the Alu subfamily inserted in exon 6B and exon 9 gene locations. In the case of exon 6B, most samples showed an expected inverted AluY insertion but we also found some cases where the exonization involved an inverted AluSc8 insertion instead (Figure 9). Because of the high similarity of the consensus sequences of AluY and AluSc8 (98.4% sequence identity) and the fact that AluSc8 subfamily is thought to be the evolutionary progenitor of the younger (Y) Alu subfamily [59,60], we hypothesize that the two different results obtained is due to an incorrect prediction made by the algorithm as opposed to TE insertion variability in this location. Additionally, the AluSc8 insertion was only observed in sequences obtained from the 1000 Genomes Project database. In other words, this AluSc8 insertion may be due to low sequencing read depth that is characteristic of the 1000 Genomes Project samples.

**Figure 10:**
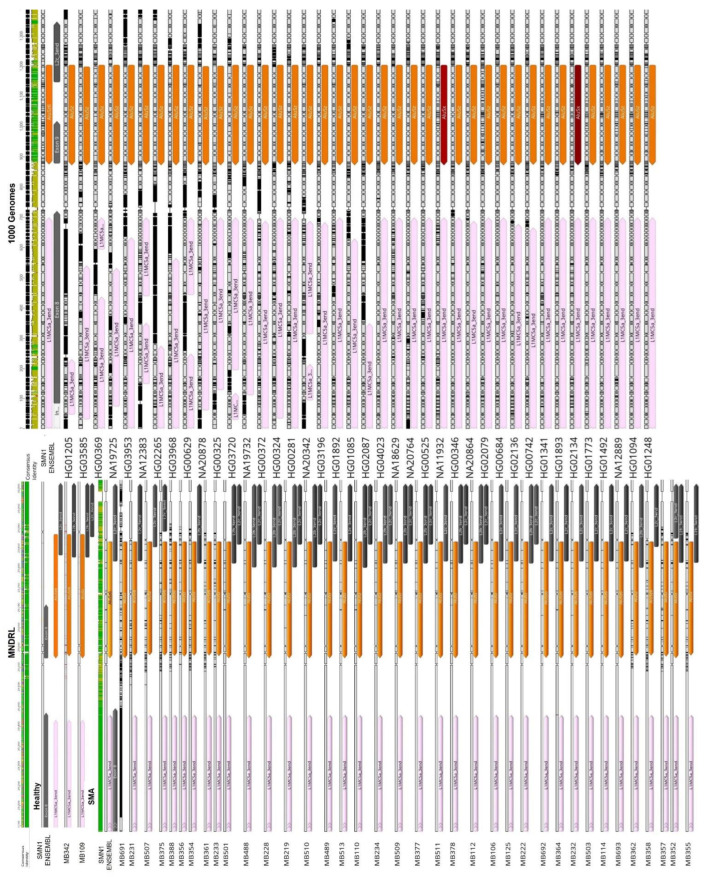
Comparison of the exon 9 region within the sample cohort. Extensive insertional polymorphisms were detected for exon 9 region in the analyzed samples. While the expected AluSz insertion (represented by a light orange arrow) was present in some samples, other AluSz6 and AluSx insertions (represented by an orange arrow and a dark red arrow, respectively) were observed in this region. This polymorphism may be due to interpersonal variability and is not associated with SMA.

Extensive insertional polymorphisms were also detected for exon 9 in the analyzed samples. Instead of the expected AluSz TE as described by [58], we observed AluSz6 and AluSx insertions in addition to the expected AluSz, as described (Figure 10). Since there is no clear connection between this polymorphic insertion and SMA disease state, we conclude that the polymorphism of this insertion may be due to interindividual variability.

### 3.3. Transposable Elements and Partial Deletions of SMN1

**Figure 11:**
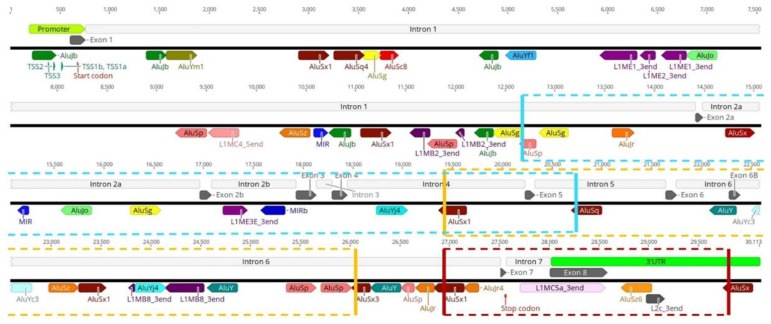
Identification of Alu-mediated partial deletions within *SMN1*. The several Alu-derived repeats existent in *SMN1* introns provide a fertile source of Alu/Alu recombination events, known to lead to gene deletions. To date, three Alu-mediated deletion events were reported in *SMN1*. The more common deletion involving exon 7 and 8 is represented by a red box. A deletion event first described by Wirth et al. [37] involving exons 5 and 6 is indicated by a yellow box. Lastly, the more recently reported Alu-mediated deletion in *SMN1* is highlighted by a blue box.

The most direct link between activity of TEs and SMA onset stems from their ability to mediate recombination events that are known to lead to disease-associated deletions and other genomic rearrangements [8,61]. The presence of several Alu repeats within *SMN1*/*2* that are in close proximity to each other, as previously discussed, make these genes particularly prone to Alu/Alu recombination events. The first Alu mediated deletion reported in *SMN1* is a deletion involving a large sequence of the gene from intron 4 to intron 6, involving exons 5 and 6 [37]. Our analysis of this breakpoint revealed that an AluSx1 element located in intron 4 and an AluSx3 in intron 6 are the Alu subfamilies responsible for the recombination event that led to exons 5 and 6 deletion (Figure 11; yellow box). Ruhno and colleagues [36] recently reported a partial deletion of the critical exons 7 and 8 whose breakpoints were within the Alu-rich intron 6 and the gene 3′UTR [36]. Our analysis revealed an AluSx1 element in intron 6 directly upstream of exon 7-and an AluSx insertion in the 3′UTR region downstream of exon 8 (Figure 11; red box). An Alu/Alu recombination event was reported in a SMA patient with a deletion of SMN1 exons 2A, 2B, 3, 4 and 5 [38]. Our analysis of this deletion event showed that an antisense AluSp of intron 1 is the most likely element to be involved in the recombination event with the antisense AluSq of intron 5 (Figure 11; blue box).

## 4. Discussion

In this work we have found that the sequences of the *SMN1*/*2* genes are enriched in TE insertions, including in key gene regions. These insertions may have important effects on regulation, splicing, expression and overall stability of the genes. TE insertions in the 5′UTR and promoter region of protein coding genes are common events with whole-genome analyses showing that up to 25% of human genes have TEs in their promoter and/or untranslated regions [62,63,64]. SINEs in particular seem to be highly represented in these regions, owing to their higher affinity to G + C rich genome regions [63,65]. The presence of TEs in 5′ regions of genes brings an evolutionary advantage for both the TE and for the host genome. For the TE, it represents an opportunity for translation as insertion in this open chromatin environment promotes its expression, and therefore its transposition [9,66]. For the host genome, the presence of TEs in these critical regions is a potential source of novel regulatory sequences by fusing with/replacing a canonical gene promoter or alternatively serving as an alternative promoter either upstream or downstream of the canonical transcription start site [15,67,68,69].

While these domesticated TEs may be integrated in the gene regulatory network, the significance of the remaining motifs inside the AluJb sequence remains to be investigated. The TSSs present inside the Alu sequence are tissue-specific and/or developmental stage-specific TSSs, with TSS2 being used as a fetal transcription start site and the use of TSS3 is still unknown [53]. This AluJb most likely serves as an alternative promoter or even as a tissue/developmental stage-specific promoter to *SMN1*/*2*. The presence of this Alu insertion in all samples in study regardless of disease status suggests a complete domestication of this element as an alternative promoter of the genes. Therefore, this insertion has led to an increased complex regulatory network capable of altering *SMN* expression, both in cis and trans. Promoter regions harboring Alu elements are subject to regulation in trans by long noncoding RNAs (lncRNAs) [70]. Future studies will experimentally confirm the functional presence of these alternate TSSs by using rapid amplification of cDNA 5′ ends (5′RACE) and their roles in developmental stage-specific and tissue-specific regulation of *SMN* expression.

The majority of human genes use alternative polyadenylation sites that are embedded in TEs, suggesting that these can influence the 3′ end processing of host gene transcripts [71,72]. L1 and other TE insertions are capable of interfering with endogenous cis regulatory elements present in 3′UTRs by introducing miRNA binding sites, promoting RNA editing and introducing polyadenylation signals [6,11,67,73,74,75]. Polyadenylation signals contained within retrotransposon sequences often lead to truncated or elongated 3′UTRs of full-length gene transcripts by providing an alternative terminator. As a result, these TE capabilities could repress transcription from the affected gene. This observation strengthens our hypothesis that the L1 present in *SMN1*/*2* 3′UTR region serves as an alternative terminator for the genes transcription by giving rise to longer transcripts. We will experimentally confirm this hypothesis in future studies using rapid amplification of the 3′cDNA ends (3′RACE) in control and SMA cells to identify alternative 3′UTRs.

Two factors may explain how this L1 element became fixed in this gene region. First, exon 8 has a lower percentage of G + C content (36.4%) when compared with the whole gene region (42.3%). The lower G + C content may have favored an L1 insertion as these elements have a bias towards lower G + C regions of the genome [76]. Second, the fact that exon 8 serves as the 3′UTR may have facilitated insertion of the L1 element as pressure against TE insertions is often relaxed in these regions [67,77,78]. We speculate, however, that such a large L1 insertion inside the gene coding region most likely had a large impact in the gene sequence and regulation and therefore should have been under negative selection pressure. Accordingly, L1 elements are especially underrepresented within genes, particularly those in the same transcriptional direction as the gene, because of their size and interference originated by retroelement regulatory motifs such as polyadenylation signals [1,3,77]. Thus, the insertion of this L1 element in the last exon of *SMN1*/*2*, that functions as the 3′UTR of the gene, is an example of a relatively rare event. Furthermore, the L1 insertion in exon 8 does not belong to the active L1 elements (also known as “hot L1 elements”) of the human genome that are composed only of the L1PA1 and L1PA2 subfamilies [3,9]. Therefore, this insertion is likely to be fixed in the human genome and not the result of a recent transposition event.

3′UTR retrotransposon insertions reduce mRNA expression [1,67,79]. The presence of this L1MC5a element in the longer *SMN1* isoform transcripts could explain why these transcripts are less common than their shorter counterparts. Interestingly, weakly expressing genes were found to be rich in LINE insertions what can be explained by the ability of L1 elements to disrupt transcriptional elongation based on the presence of strong polyA signals in their sequences that possibly function as transcriptional terminators [80]. We argue that the alternative terminator provided by the L1 element is used less frequently than the canonical gene terminator located in exon 7 and may only be used in a tissue/time-specific manner. Accordingly, alternative UTRs that are often provided by TE insertions, can determine tissue-specific functions of mRNAs [81,82]. 3′UTR retrotransposons insertional events, such as the L1 insertion in the 3′ region of *SMN1*/*2* reported in this study, are only moderately selected against and may provide a gradual mechanism of evolution by which retrotransposons alter the expression profile and influence crucial gene networks in the human genome [55,67,83].

Additionally, it is possible that this L1MC5a element has the same transcription terminator function in *SMN2* as it shares the same TE insertional patterns in its sequence with *SMN1*. Accordingly, the longest *SMN2* transcript isoform presents this L1 insertion in its 3′ region, in an identical sequence position as in *SMN1* longest transcript. This hints to this retrotransposon having the same alternative terminator role in *SMN2* transcription to that in *SMN1* transcription. We hypothesize that this alternative terminator role can have implications in SMA severity by reducing *SMN2* mRNA expression due to the presence of this L1MC5a element within *SMN2*. The confirmation of this hypothesis will require further analysis using a combination of experimental and in silico methodologies.

Circular RNAs (circRNAs) are a widely expressed class of non-colinear RNAs generated in a diverse set of eukaryotic organisms [84]. Due to their lack of 5′ and 3′ termini, these RNAs are extremely stable meaning that even small levels of circRNAs may affect cellular metabolism by sequestering/sponging miRNAs, sequestration and trafficking of proteins, regulation of transcription and generation of short RNA-binding proteins [84,85]. CircRNAs are important regulators of cellular physiology and also potential biomarkers of disease onset or progression [84,86]. Circular RNAs have been associated with various human diseases, particularly cancer, diabetes mellitus, cardiovascular diseases, chronic inflammatory diseases and neurological disorders [84,85,87]. The most common way in which circRNAs are generated is through backsplicing in which the 5′ splice site of a downstream exon is paired with the 3′ splice site of an upstream exon [58,88]. One of the defining features of backsplicing events appears to be the RNA secondary structure formed by inverted short repeats, especially Alu elements, within intronic sequences upstream and downstream of the 3′ and 5′ splice sites [58]. The existence of several inverted Alu repeats here detected throughout the *SMN1* reference sequence explains the high levels of circularization of *SMN1*/*2* transcripts as Alu repeats located in introns 4 and 5 are especially active in this process [35,53,58]. Pairing between the longest and highly Alu-enriched regions, introns 1 and 6, could be favored by several inverted Alu repeats thus potentially favoring backsplicing between exon 6 and exon 2A [35]. Additionally, generation of circRNAs with exons 2A, 2B, 3 and 4 requires pairing of the 5′ splice site of exon 4 with the 3′ splice site of exon 2A, which is made possible by the fact that intron 1 contains numerous Alu elements that are capable of pairing with the intron 4 Alu elements [33]. In future studies, we will experimentally confirm the presence of these putative exonization events mediated by circRNAs in SMA cells using RNA sequencing [89]. The presence of these inverted repeats that are in a favorable position to pair with each other, is often associated with alternative splicing events leading to circRNA biogenesis. Given that high levels of Alu inverted repeats in *SMN1*/*2* introns may explain how these genes generate several circular RNAs, we believe that circRNA biogenesis occurs in healthy individuals and SMA patients to the same extent. It is possible, however, that dysregulation of these Alu repeats in SMA-affected genomes may lead to an increased formation of circRNAs coded by *SMN1*. These higher levels of circRNAs formation and the widespread alternative circularization of *SMN1*/*2* pre-mRNA may have a still undiscovered role in SMA onset or may contribute to worse SMA phenotypes, owing to circRNAs ability to interfere with the coding capacity of human genes [35,58,84]. Additionally, circRNA formation in *SMN1*/*2* may function as a potential biomarker for the genes’ overall transcriptional/splicing stability since higher circRNAs levels indicate aberrant RNA splicing events that may be linked to SMA.

The two exonization events within the *SMN* genes, exon 6B [56,57] and exon 9 [58], have been generated by different Alu insertions. All known Alu recombination events that led to deletions of *SMN1* occurred among Alu elements of the (S) subfamily supporting the idea that sequence identity between the two elements at a *locus*—alongside proximity—appear to be proportional to their chances of successful recombination [61,90,91]. The high conservation of position and orientation of the Alu insertions involved in these exonization events implies that these domestication events occur identically in healthy and diseased genomes. Furthermore, they are important sources of novel exons that increase the coding capacity of the genes beyond the coding capacity of *SMN1*/*2* canonical exons. In a wider spectrum, Alu and other TEs provide transcriptome diversity and ultimately result in the diversification of the human proteome.

Some insertional polymorphisms that were observed in these Alu repeats between the analyzed samples may be the result of normal interpersonal sequence variability. Accordingly, polymorphic Alu elements account for 17% of structural variants in the human genome, clearly establishing a link between individual TE polymorphisms and human genetic variation [92]. Since the SMA samples here analyzed are from individuals lacking the *SMN1* gene, we cannot draw definitive conclusions about the involvement of Alu elements in the deletion events in a disease context. Our results show that in the analyzed SMA patients and in the remaining samples, the critical Alu elements responsible for these deletions are also present in the same position and orientation in the *SMN1*/*2* sequence as in the gene reference sequence and as described previously [36,37,38]. Although these Alu insertions sites are most probably the reason for the complex deletion events, the high conservation of orientation and of subfamily type found in all the samples analyzed (healthy and diseased) implies that their presence *per se* is not the reason for the deletion events. Their presence in *SMN1*/*2* introns is a source of sequence homology that can be responsible for genomic rearrangements and consequently disease in some genomes [8,61]. TE recombination may be responsible for the approximately 2% of SMA cases that result from *de novo* mutations in *SMN1* and are not inherited from carrier parents [27]. It is likely that the several Alu repeats present throughout *SMN1* are responsible for *de novo* deletions in germinative cells due to unequal recombination since TE silencing mechanisms are often relaxed in these developmental stages [3,9,19]. Alu elements may indeed, play an important role in the high instability of the *SMN1*/*2* genomic region leading to disease-causing deletions of *SMN1* exons and potentially whole gene deletions under specific circumstances. Alu elements invasion of *SMN1*/*2* makes these genes very susceptible to Alu-mediated deletions, that have critical consequences to genome stability and host health.

## 5. Conclusions

Our analysis of the *SMN* genes revealed a pervasive invasion of its sequence by TEs that we believe may severely impact these genes’ regulation structure, expression and overall genomic stability. The several TEs present inside these genes, especially Alu and L1 elements that are highly enriched in the promoter and intronic regions of the gene, seem to play important roles in gene expression, novel exon creation, alternative splicing and deletion events known to lead to SMA. Additionally, a L1 element insertion in the 3′UTR region of the gene is also responsible for a domestication event that gave the gene an alternative terminator, therefore increasing the diversity of *SMN1* transcripts and being a prime example of how a TE insertion inside a protein-coding gene can create a gradual mechanism of evolution by which retrotransposons alter the human transcriptome.

The in silico analysis of *SMN1*/*2* completed in this work serves as a starting point for further investigations on the impacts of TEs in human disease and particularly, their role in SMA onset and severity. While the TEs identified in this in silico analysis were present in both *SMN1* and *SMN2,* it is possible that they may affect the regulation of these genes differently. Future studies will further characterize the effects of these TEs in *SMN1* and *SMN2* on gene regulation under healthy conditions as well as in SMA.

## Figures and Tables

**Table 1:** 1000 Genomes Project sample cohort.

Sample ID	Carrier Probability	Carrier Status
HG02134	1	Carrier
NA12383	1	Carrier
HG01773	1	Carrier
HG00346	1	Carrier
HG00281	1	Carrier
HG02087	1	Carrier
HG01085	1	Carrier
HG01893	1	Carrier
HG00324	0.997	Carrier
NA20764	0.982	Carrier
HG02265	0.982	Carrier
HG02079	0.976	Carrier
HG03953	0.972	Carrier
HG01248	0.935	Carrier
HG01492	0.914	Carrier
HG01892	0.902	Carrier
HG00525	0.763	Carrier
HG01205	0.756	Carrier
HG01094	0.738	Carrier
NA11932	0.716	Carrier
HG00629	0.000165	Non-Carrier
HG03585	0.000159	Non-Carrier
HG01341	0.000155	Non-Carrier
HG00325	0.000151	Non-Carrier
HG00369	0.000134	Non-Carrier
NA20878	0.000131	Non-Carrier
HG04023	0.000127	Non-Carrier
HG02136	0.000126	Non-Carrier
NA19732	0.000126	Non-Carrier
HG00684	0.000126	Non-Carrier
NA18629	0.000126	Non-Carrier
NA20864	0.000122	Non-Carrier
HG03196	0.000112	Non-Carrier
HG00372	0.000111	Non-Carrier
NA12889	0.0000997	Non-Carrier
NA19725	0.0000982	Non-Carrier
HG03720	0.0000969	Non-Carrier
HG00742	0.0000923	Non-Carrier
NA20342	0.0000907	Non-Carrier
HG03968	0.0000851	Non-Carrier

**Table 2:** Phenotypic and copy number information for samples within the MNDRL Cohort.

		Copy Number (Exon 7)		Copy Number (Exon 8)	
Sample ID	Phenotype	*SMN1*	*SMN2*	*SMN1*	*SMN2*
MB109	healthy	3	1	2	1
MB342	healthy	2	0	2	0
MB106	SMA II	0	3	0	3
MB110	SMA I	0	3	0	3
MB112	SMA II	0	3	0	3
MB114	SMA I	0	2	0	2
MB125	SMA III	0	3	0	3
MB219	SMA I	0	2	0	2
MB222	SMA II	0	3	0	3
MB230	SMA III	0	3	0	3
MB231	SMA III	0	4	0	4
MB232	SMA II	0	3	0	3
MB233	SMA I	0	3	0	3
MB234	SMA I	0	2	0	2
MB352	SMA II	0	3	0	3
MB354	SMA II	0	3	0	3
MB355	SMA II	0	3	0	3
MB356	SMA III	0	4	0	4
MB357	SMA II	0	3	0	3
MB358	SMA II	0	3	0	3
MB361	SMA II	0	3	0	3
MB362	SMA I	0	2	0	2
MB364	SMA I	0	2	0	2
MB375	SMA II	0	3	0	3
MB377	SMA III	0	4	0	4
MB378	SMA II	0	3	0	3
MB388	SMA III	0	3	0	3
MB488	SMA I	0	2	0	2
MB489	SMA I	0	2	0	2
MB501	SMA I	0	2	0	2
MB503	SMA III	0	4	0	4
MB507	SMA I	0	2	0	2
MB509	SMA I	0	2	0	2
MB510	SMA I	0	2	0	2
MB511	SMA II	0	3	0	3
MB513	SMA III	0	3	0	3
MB691	SMA I	0	2	0	2
MB692	SMA I	0	2	0	2
MB693	SMA I	0	2	0	2

## Data Availability

The datasets analyzed during this study are available from Butchbach (matthew.butchbach@nemours.org) on reasonable request.
